# Inherited neurometabolic diseases and the importance of imaging-based classification systems

**DOI:** 10.1590/0100-3984.2022.55.3e2-en

**Published:** 2022

**Authors:** Nina Ventura

**Affiliations:** 1 Instituto Estadual do Cérebro Paulo Niemeyer, Rio de Janeiro, RJ, Brazil; Universidade Federal do Rio de Janeiro (UFRJ), Rio de Janeiro, RJ, Brazil; Hospital Samaritano, Grupo Fleury, Rio de Janeiro, RJ, Brazil

Inherited neurometabolic disorders (INMDs) constitute a heterogeneous group of diseases that includes the so-called inborn errors of metabolism, leukodystrophies, mitochondrial diseases, and deposition diseases^(1)^. Although INMDs are rare individually, they collectively account for a significant proportion of neurological diseases in children^(1,2)^. The clinical and imaging findings of INMDs can be nonspecific and can overlap, making the diagnosis challenging. However, because there are treatments available for some INMDs, early diagnosis is fundamental in order to minimize or prevent neurological damage^(1)^. Therefore, it is essential that neuroradiologists are able to recognize the imaging findings that can narrow the differential diagnosis.

Magnetic resonance imaging is the modality of choice for the evaluation of various diseases of the central nervous system^(3-7)^, including INMDs, being crucial for the localization, identification, and characterization of alterations. It is still the main method employed for monitoring patients with INMDs, either to evaluate the natural progression of the disease or to assess the response to treatment, as well as being used in order to screen family members for genetic counseling^(8-10)^.

Over the years, a number of classifications based on magnetic resonance imaging findings have been proposed, in attempts to facilitate the approach to INMDs and demystify the topic. Recent articles have proposed divisions by disease groups, such as leukodystrophies or mitochondrial diseases^(10,11)^, or an epidemiological division, with separate categories for disorders that occur only in adults or children^(8,12)^. Some authors have also shown that specific imaging findings, such as the pattern of involvement of the white matter and basal ganglia, as well as the presence or absence of cysts and a T2-weighted signal in the corpus callosum, are quite useful for guiding the diagnosis^(9,13)^.

The article authored by Pedri et al.^(14)^, entitled “Classification of inherited neurometabolic disorders based on radiological aspects: pictorial essay” and published in the previous issue of Radiologia Brasileira, proposes a new classification system that encompasses all INMDs, of any epidemiology or subtype. That system is easily applied in daily practice because it is based on the patterns seen on diagnostic imaging. The authors identified 10 distinct patterns: macrocrania; cysts; distribution of white matter involvement; contrast enhancement; calcifications; basal ganglia involvement; restricted diffusion; vascular abnormalities; cranial nerve involvement; and the metabolic profile on spectroscopy. The article presents practical divisions and could be an excellent resource when we encounter any suspicion of an INMD.

Despite the fact that INMD is a complex subject that is avoided by many radiologists, it is inevitable that we will come across some cases over the course of our careers. Knowledge of the key findings and imaging patterns can facilitate the investigation and make the diagnosis of INMDs less challenging, as can reliable, concise sources for consultation with practical guides, such as that provided by Pedri et al.^(14)^.
